# Addressing Food and Nutrition Security in Developed Countries

**DOI:** 10.3390/ijerph16132370

**Published:** 2019-07-04

**Authors:** Christina Mary Pollard, Sue Booth

**Affiliations:** 1 Faculty of Health Science, School of Public Health, Curtin University, GPO Box U1987, Perth 6845, Australia; 2 College of Medicine & Public Health, Flinders University, GPO Box 2100, Adelaide 5000, Australia

**Keywords:** food security, food insecurity, social assistance, poverty, homeless, nutrition environment, food stress, food affordability, policy, intervention, determinants, food banks, developed countries

## Abstract

The guest editors of the special issue on *Addressing Food and Nutrition Security in Developed Countries* reflect on the 26 papers that were published as part of this issue and the scope of research contained therein. There is an extensive body of work, which focuses on topics ranging from the prevalence of food insecurity in developed countries, associations and determinants, measurement and monitoring, to reports of the lived experience and coping strategies of people who are living with food insecurity or and those who are a part of the charitable food sector. Very few solutions to address the problem of food insecurity in developed countries were offered, and many challenges highlighted. Further research is required to find the solutions to address the problem of food insecurity in developed countries, and important principles and values are proposed for those undertaking this work to embrace.

Improving food and nutrition insecurity has become a public health priority in developed and economically rich countries, such as Australia, Europe, the United Kingdom, Canada, and the U.S. [1]. Food insecurity is costly and has wide-reaching consequences, with its effects extending beyond vulnerable populations. For example, for women residing in high-income countries, food insecurity is associated with an increased risk of depression while conversely, depression is also a predictor of food insecurity [2]. Pregnant women and mothers; women at risk of or are experiencing homelessness; refugees; and those exposed to violence and substance abuse were at the highest risk [2]. In Canada, any experience of food insecurity in both males and females was associated with adverse mental health outcomes [3]. The prevalence of food insecurity among Asian Americans was highest among Vietnamese and lowest among Japanese subgroups and varied by acculturation [4].

Understanding the factors associated with food insecurity can assist in identifying effective responses. Analysis of the 2014 General Social Survey of the Australian population quantified the association between 18 discreet stressful life events and food insecurity. Stressors related to employment and health doubled the likelihood of experiencing food insecurity [5]. Household food insecurity was also associated with receipt of specific social assistance payments in Australia, suggesting that these families were enduring significant financial stress [6]. It is not just welfare-dependent households who are experiencing food insecurity in Australia, the prevalence is increasing in low to middle income groups [7,8]. The complex and interactive nature of the factors associated with food insecurity has also been quantified in one Australian study [8]. Researchers highlighted the need for comprehensive policies and programs that recognize the complex links with other social and public health challenges [1,2,5,9] and recommended the adoption of both nutrition-sensitive and nutrition specific interventions [1].

Long-term food insecurity after disasters is another concern for sociodemographically challenged populations in developed countries. Examination of the impact of Hurricane Katrina on families five years after the event found that higher income, race, and having a partner were protective factors against food insecurity whereas low social support, poor physical and mental health, and being female were risk factors [10].

As governments retreat from the issue, private charitable and not-for-profit sector organizations step in to deliver food and other services to people in need. This is particularly evident in the rapid expansion and proliferation of food banks and charitable food services. In 2017, the population access to Tafel food banks in Germany was such that nearly all residents, including welfare recipients, have access to at least one food bank located in their local district [11]. Public and political debate is continuing about the appropriateness of food banks as the main response to food insecurity in developed countries [1,11].

## 1. The Experiences of Food Insecurity in Developed Countries and Coping Strategies

Australian Aboriginal and Torres Strait Islander people are significantly more likely to experience food insecurity than their non-indigenous counterparts, particularly those residing in rural or remote areas. There is limited evidence regarding the prevalence of food insecurity among families with young children residing in urban areas. For these families, food insecurity usually occurred intermittently and due to the unaffordability of food relative to income and living expenses, resulting in limited food choice and poorer meal quality. Family support, the main coping strategy, should be considered as an essential safety net in public policy to address food insecurity [12].

The perceptions of frontline service providers on the nature of food insecurity also provide insights on effective interventions. In Scotland, a country-wide study of informants from twenty-five health, social care, and third sector organizations was undertaken. Food insecurity was described as having multiple faces and related factors with concerns being raised regarding those at risk of food insecurity, including working families, young people and women. The difficulty in accepting external help was aptly described as ‘stoicism and struggle’. The pessimistic view of the participating community regarding the needs of food insecure groups is of great concern [13]. Australian low- to middle-income families describe similar tensions as they struggle to balance a range of financial, social, physical and personal assets to avoid or alleviate the experience of food insecurity [7].

## 2. Measurement and Monitoring

Measuring and monitoring of food insecurity and its determinants is a salient concern in some economically developed nations. The absence of robust food insecurity monitoring and surveillance systems in the households of Australia, Scotland and Europe has led researchers to undertake a secondary analysis of related surveys, such as Scotland’s Living Costs and Food Survey [14] and Australia’s Household Expenditure Survey [6], in order to determine the nature and prevalence of household food insecurity [15]. Food affordability, a key component of food security, has been determined using comparisons of the weekly food expenditure and its ratio to equalized income for households with varying income levels in Scotland [14] and Australia [16,17]. Analysis trends in the relationship between food affordability at the household level and diet quality in Scotland found that poorer households were less likely to achieve recommended dietary intakes over time [14]. However, the authors concluded that robust and comprehensive systems are needed to provide the full picture. Across Europe, a Food Reference Budget has been developed to contribute to the prevention of food insecurity in low income contexts [15].

The same questions are being asked in Australia and New Zealand in the absence of robust and comprehensive food insecurity monitoring systems. Similar to the concept of rental stress, the innovative geographically based Food Stress Index was developed using the Western Australian Government’s Food Access and Cost Surveys and relevant sociodemographic census data to determine place-based risk of food stress [16]. Emergency relief service providers and government policy makers are very interested in applying the FSI to identify areas of particular need for food security action.

The Healthy Diets ASAP (Australian Standardized Affordability and Pricing) method assesses the affordability of healthy (recommended) and contemporary (unhealthy) diets. In rural Australia, the price of the contemporary (unhealthy) diet was shown to be more expensive than the recommended healthy diet [18]. Furthermore, a tailored Aboriginal and Torres Strait Islander version tested in five remote communities found similar results, with food alarmingly found to not be affordable in either of these areas [17]. A version was also developed for the contemporary New Zealand diet and assessed with consideration of their dietary recommendations. Expert panels assisted in tailoring the instrument for different population subgroups, including Māori and Pacific households, with the healthy diet again found to be more affordable [19]. The nutritional environment can influence the availability and accessibility of food, which are both components of food insecurity. Nutrition environment measurement tools were applied and they found that in rural and socially disadvantaged communities in Australia, it is harder to access nutritious food at affordable prices [20].

## 3. Perspectives of Charitable Food Sector and Food Banking Staff and Recipients

The lived experiences of Finnish food aid recipients debunks public perceptions that people are somehow responsible for their own poverty and highlighted the worsening income insufficiency, deepening poverty and the inability of aid agencies to cope [21]. The same phenomena is occurring in Australia where charitable food services persevere with limited resources [22]. There is emerging evidence that traditional food assistance models further stigmatize people and are inadequate. Australian research sought the perspective of users on existing services and ideas for improved models. Empowering and dignified food assistance models that enable choice and reciprocity provide opportunities for social interaction and connection, with links to broader supports being strongly recommended [23].

## 4. Solutions to Address the Problem of Food Insecurity in Developed Countries

There are limited examples of interventions that are effective in reducing food insecurity in developed countries. Monetary incentives to encourage fruit and vegetable purchases in remote Aboriginal communities show limited success due to the multiple challenges related to the operational running of the community stores, but were highly valued by women with children and accepted by the community [24]. Examination of the decision-making processes of remote community store owners, retailers, and health promotion professionals highlighted the importance of involving store owners and policy makers in the design of interventions [25].

Clearly, further research is required to develop effective interventions to address food insecurity in developed countries. Discrimination, academic expectations, siloed thinking, and cultural differences are some of the challenges to sharing research expertise that must be overcome [26]. Principles and values that can help to drive potential solutions to address these research challenges have been proposed, along with a call for the international research community to adopt them [26].
